# Effect of digital complete dentures manufactured using the custom disk method on masticatory function^☆^

**DOI:** 10.1016/j.heliyon.2023.e23938

**Published:** 2023-12-17

**Authors:** Maiko Iwaki, Manabu Kanazawa, Yumika Soeda, Tamaki Hada, Yuriko Komagamine, Shunsuke Minakuchi

**Affiliations:** a Digital Dentistry, Graduate School of Medical and Dental Sciences, Tokyo Medical and Dental University, Tokyo, Japan; b Gerodontology and Oral Rehabilitation, Graduate School of Medical and Dental Sciences, Tokyo Medical and Dental University, Tokyo, Japan

## Abstract

**Statement of problem:**

The effect of using the custom disk method (CDM) for fabricating digital dentures on patients’ masticatory function should be studied to support its use in clinical practice.

**Purpose:**

To investigate the effect of digital dentures fabricated using CDM on patients’ masticatory function.

**Material and methods:**

This single-center prospective clinical study included 20 patients with edentulous maxillary and mandibular arches who used a complete denture. The digital impression and complete denture manufacturing procedures using CDM have already been reported by Kanazawa et al. (2018) [32] and Soeda et al. (2022) [18] Thedigital dentures fabricated with CDM were delivered to the participants, and periodic adjustments were made until the patient could use the denture without pain. A color-changeable chewing gum, two types of gummy jellies that can evaluate the masticatory function, and pressure-sensitive sheets were used to evaluate the participants’ masticatory function at baseline, 1 month, and 6 months following adjustment of the new digital complete dentures fabricated with CDM. These masticatory function values had already been measured in the previous conventional dentures and were recorded as baseline values.

**Results:**

The study participants included 8 women and 12 men (mean age, 77.6 years). The color-changeable chewing gum analysis indicated that there was no significant improvement of masticatory function from baseline to 1 M (P = .083) and 6 M (P = .157).The gummy jelly analysis indicated no significant differences between the masticatory function baseline and 1 month (P = .387); however, a significant improvement was observed from baseline to 6 months (P = .020). Tests with Glucolum indicated a significant improvement from baseline to 1 month (P = .012) and 6 months (P = .003). The maximum bite force and occlusal contact area showed no significant difference at any time point.

**Conclusions:**

Significant improvement in masticatory function was observed upon evaluation with gummy jelly and Glucolum 6 months after delivering the new digital complete dentures. Under limited conditions, the digital denture fabricated using CDM resulted in good recovery of the masticatory function in elderly edentulous patients. The present results combined with the cost-effectiveness and patient satisfaction associated with CDM indicate its clinical utility.

## 2 Introduction

In recent years, complete dentures (CDs) are being increasingly fabricated using digital technology, especially in the aging society, which is expected to effectively improve denture quality and accuracy [[1], [2], [3], [4]]. Digital CDs can be classified as milled [[5], [6], [7]] and three-dimensional (3D) printed dentures [[8], [9], [10]]. A milled denture guarantees excellent mechanical properties because the denture is milled from a cured resin disk [11] that does not undergo resin polymerization or shrinkage. It thus provides a more accurate and reproducible fit on the mucosal surface [6,7], with less displacement of artificial teeth than that in conventionally-fabricated dentures [12]. Digital CDs not only provide high patient satisfaction [13,14] but also reduce labor costs [14] and chairside time; further, the total cost of denture fabrication can be reduced by using machines to perform most of the technical work [15]. However, the effect of digital CDs on patients’ oral health-related quality of life is reportedly equivalent to that of conventionally-fabricated CDs [16].

Until now, the most common method of manufacturing milled dentures has involved bonding of readymade artificial teeth to a machined denture base using computer-aided design and computer-aided manufacturing (CAD/CAM) [1]. However, this method causes problems with the adhesive portion of the artificial tooth and denture base [17], as well as misalignment of the artificial teeth [1]. Therefore, in recent years, methods have been developed to simultaneously mill the denture base and artificial teeth [14,17]. In addition, the custom disk method (CDM) has been introduced, in which dentures are produced by fabricating and milling a resin disk embedded with artificial teeth aligned for each individual patient [18]. The advantages of CDM over the conventional method of fabricating digital dentures, in which artificial teeth are later bonded to a milled denture base, include improved adhesive strength between the artificial tooth and denture base and superior esthetics [19]. Since CDM significantly improved patient satisfaction and significantly lowered labor and total costs compared to CD, the cost-effectiveness analysis using incremental cost-effectiveness ratio (ICER) proved that CDM was more cost-effective than CD [20].

The masticatory function of CD wearers is reduced to 1/2–1/6th of that with natural teeth [21,22]. Approximately 30 % of these patients are unsatisfied with their dentures and experience issues [23], such as difficulty in chewing hard foods, denture pain and instability, and malnutrition [[24], [25], [26]]. A weak correlation between masticatory function and denture satisfaction has been observed [27], and quantitative evaluation of masticatory function can help to effectively determine the success of a prosthetic device [28,29]. A previous study showed no improvement in the masticatory function of patients who received a new CD prepared using the conventional method [30]. A randomized controlled trial showed that the chewing efficacy of patients with old conventionally-fabricated dentures improved on placing new dentures prepared with a commercial digital denture system; however, the maximum bite force did not improve [31].

Therefore, this study aimed to evaluate the masticatory function of patients with digital CDs fabricated using CDM and examine the effect of CDM. The null hypothesis of this study was that masticatory function with CDs fabricated using CDM does not differ from that with conventionally-fabricated CDs at baseline.

## 3 Materials and methods

This single-center prospective clinical study was approved by the Ethical Review Committee of Tokyo Medical and Dental University (registration number: D2017-016) and registered with the University Hospital Medical Information Network Center (UMIN-CTR Clinical Trial, Unique trial Number: UMIN000027708).

Twenty individuals with edentulous maxillary and mandibular arches, who had been using CDs and had no orofacial pain, temporomandibular disorders, xerostomia, and parafunctional habits, were included in this study. The exclusion criteria were severe alveolar crest or jaw bone defects, difficulty undergoing dental treatment owing to the general condition, inability to communicate due to dementia or other reasons, and inability to visit the hospital. Participants were recruited from among patients who visited the Department of Dentistry, Tokyo Medical, and Dental University Hospital. All participants provided written informed consent.

**Fig. 1:**
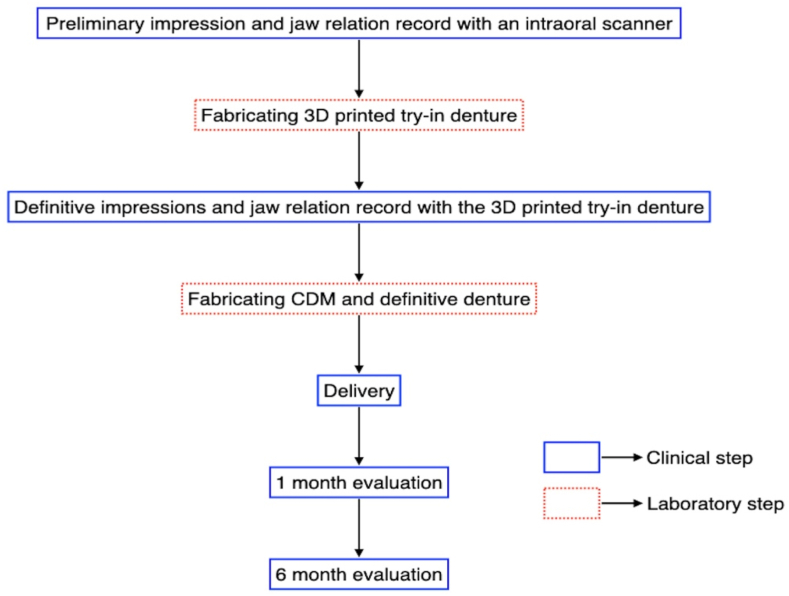
Denture fabrication and evaluation procedures.

Fig. 1 shows the denture fabrication and evaluation procedures. All clinical procedures for denture fabrication were performed by three dentists with 10–15 years of clinical experience in the Department of Prosthodontics of the university hospital, two of whom (M.I. and M.K.) are board-certified by the Japanese Society of Prosthetic Dentistry. The clinical procedures for digital impressions and CD manufacturing using CDM have already been reported by Kanazawa et al. [32] and Soeda et al. [18].

**Fig. 2:**
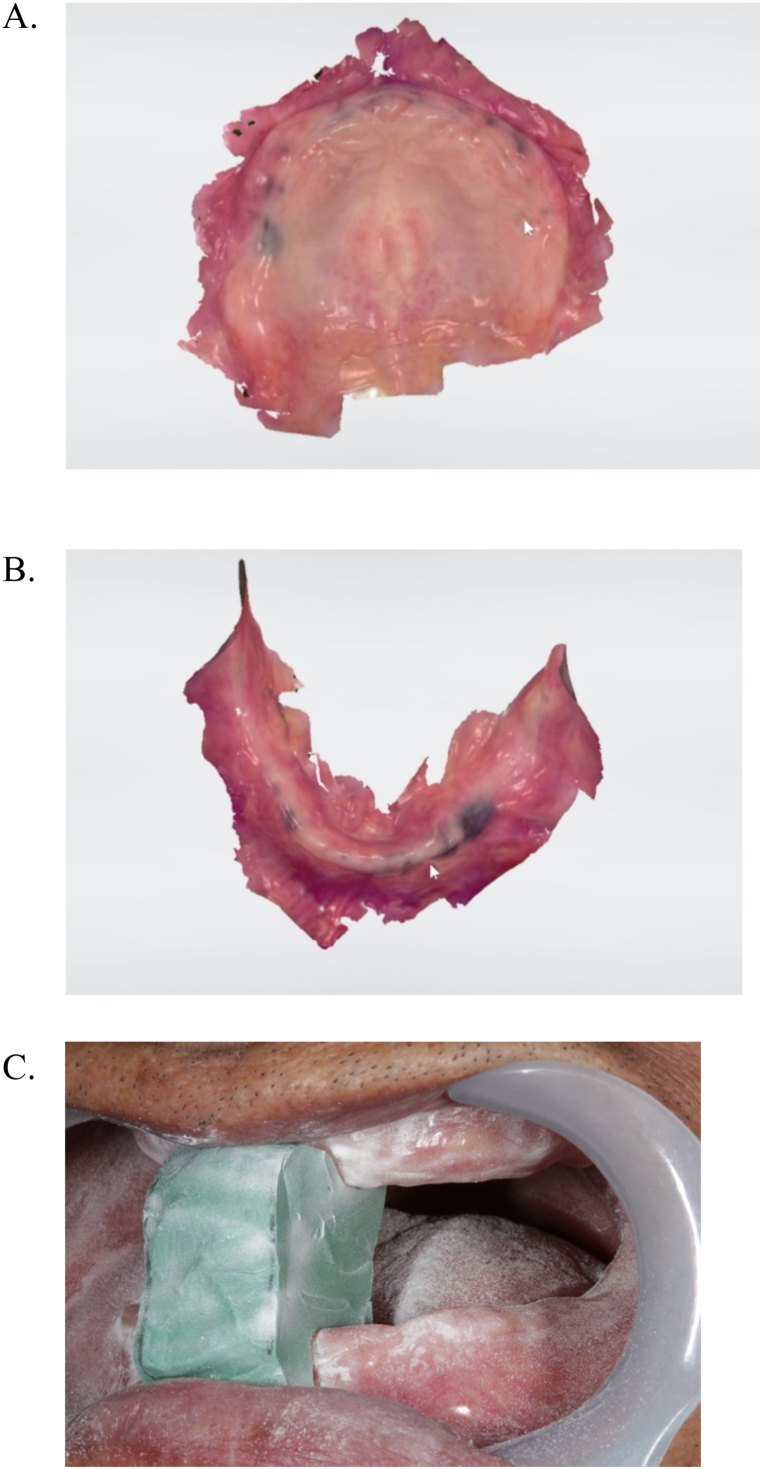
The edentulous maxillary and mandibular arched were scanned using an intraoral scanner. A, Scanned data of maxilla. B, Scanned data of mandible. C, Jig was placed between maxillary and mandibular ridges and scanned using intraoral scanner to record intermaxillary relationship.

At the first visit, the edentulous maxillary and mandibular arched were scanned using an intraoral scanner (IOS; TRIOS3; 3Shape A/S). The scanned data were saved as separate standard tessellation language (STL) files for the maxillary and mandibular arches (Fig. 2A and B). Next, a mark was placed on the patient's nose and tip of the jaw, and the distance between the marks was measured anatomically or physiologically with a caliper, upon referring to the occlusal height of the present denture, to determine the approximate occlusal height. Silicone putty impression material (FUSION II PUTTY TYPE; GC) was polymerized between the maxilla and mandible of the patient while maintaining the occlusal height. The polymerized silicone putty was cut into approximately 15-mm thick pieces and used as a jig to record the intermaxillary relationship. The jig was placed between the maxillary and mandibular arches and scanned together with the arches using an IOS to obtain a preliminary jaw relation record (Fig. 2C). The scanned data were saved as STL files. The STL data of individual maxillary and mandibular arches and of both arches with the silicone putty were imported into a CAD software (3Shape Dental System; 3Shape A/S, Geomagic Freeform; 3D SYSTEMS). The intermaxillary relationship was reproduced, and the trial dentures were designed in the software. 3D printed try-in dentures (Form3; Formlabs Inc.) were fabricated using light-cured resin (Dental SG; Formlabs Inc.) based on the virtual design.

**Fig. 3:**
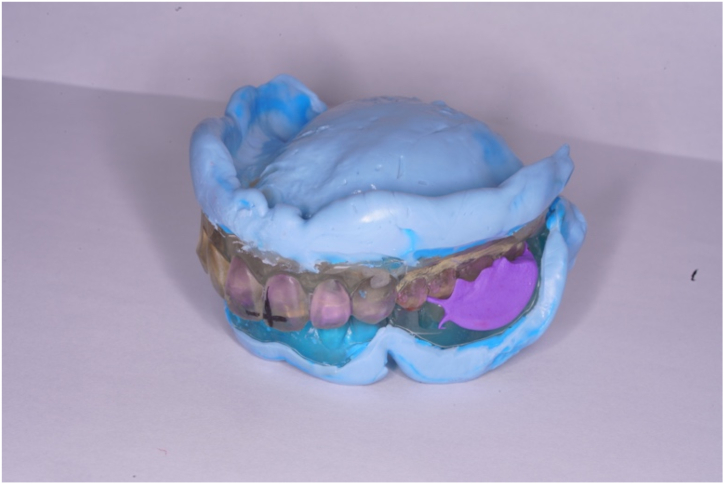
The final impression using 3D printed try-in dentures.

At the second visit, the arrangement of the anterior artificial teeth arrangement on the 3D printed dentures was evaluated. Definitive impressions (Exadenture; GC Corp) of the maxillary and mandibular arches were made, and intermaxillary records (Correct Quick Bite; PENTRON) were obtained with the 3D printed try-in dentures (Fig. 3). The impressions with the 3D printed try-in dentures were scanned with a laboratory scanner (E−3; 3Shape), and the final CDs were designed using the STL data of the final denture space by CAD software (3Shape Dental System; 3Shape A/S, Geomagic Freeform; 3D SYSTEMS).

**Fig. 4:**
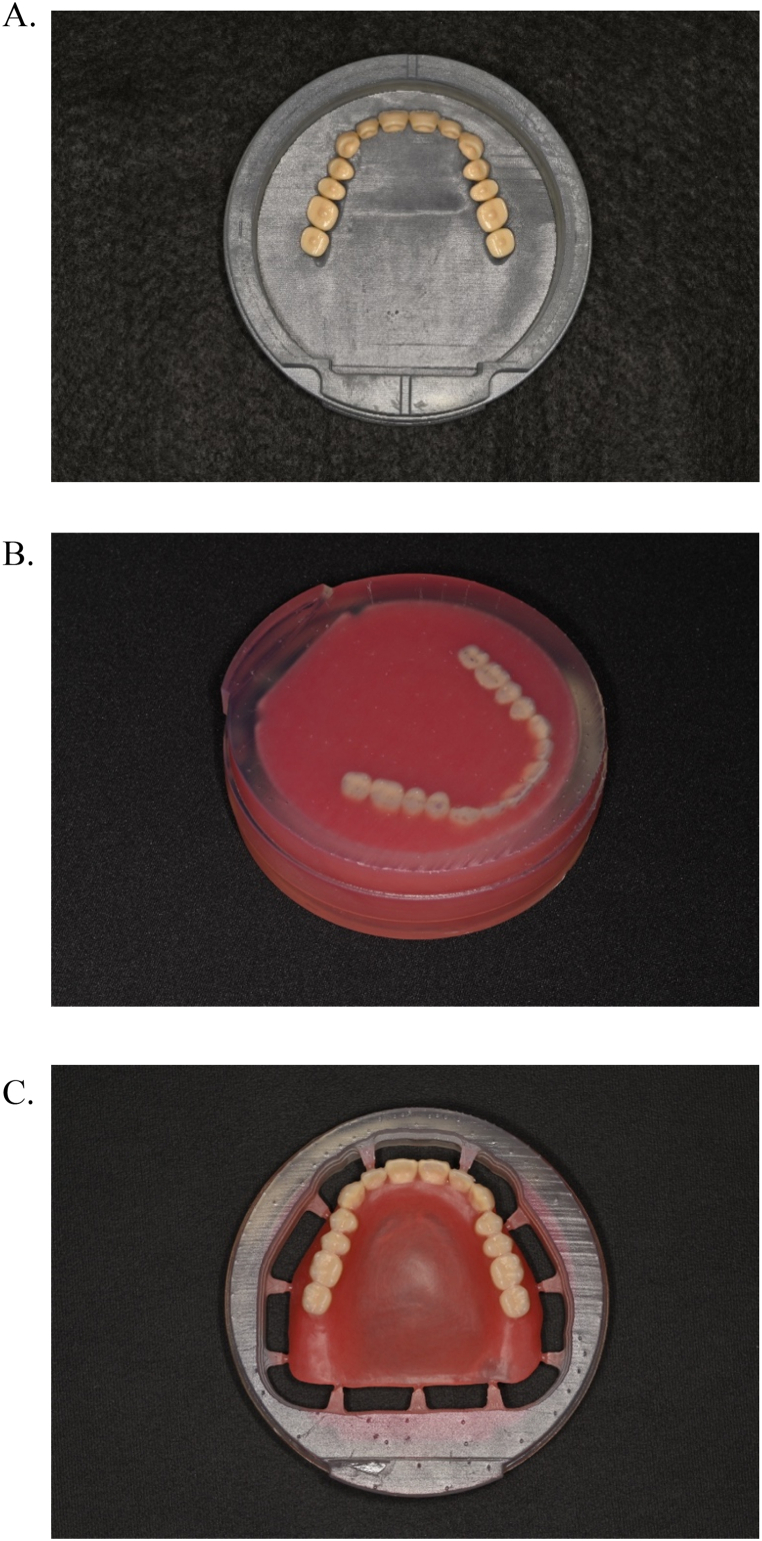
The fabrication of the custom disk. A, Artificial teeth were aligned on printed frame and bonded with instant adhesive (Aron Alpha A; Daiichi Sankyo). B, Autopolymerizing resin (Fitresin; Shofu) was poured in printed frame. C, Custom disk, including denture base and artificial teeth, was milled.

The frames of the custom disks for the maxillary and mandibular arches were designed. The 3D positioning information was incorporated in the frames to enable precise mounting on the milling machine [18]. The denture data were assembled into a custom disk data-of-frame. Next, the denture data were moved 0.1 mm in the direction toward the base of the denture along the Z-axis to compensate for potential errors due to placement of the artificial teeth in the recess. The offset value was set to 0.2 mm. Boolean logic operations were used to shape the recess and subtract the artificial teeth from the frame of the custom disk. From the STL data of the finalized frame design, a custom disk frame was 3D printed (Form 2; Formlabs). Artificial teeth were aligned on the printed frame, bonded with instant adhesive (Aron Alpha A; Daiichi Sankyo), and autopolymerizing resin (Fitresin; Shofu) was poured (Fig. 4A and B). The fabrication of the custom disk was completed by polymerizing it in a dental polymerizer (Fitresin multicure; Shofu). Using an alignment device, the custom disk was mounted on the milling machine and milled according to the designed denture data (Fig. 4C). After trimming, the denture was finished and polished in the same way as a conventional CD.

The completed digital dentures were delivered to the patient at the third visit following the usual procedure. After placing the denture, periodic adjustments were made until the patient was able to use the denture without pain. The masticatory function of the dentures was evaluated at 1 month (1 M) and 6 months (6 M) after adjustment.

To evaluate the masticatory function, a color-changeable chewing gum (Xylitol Masticatory Performance Evaluating Gum; Lotte Co.), two kinds of gummy jellies that can evaluate the masticatory function, and pressure-sensitive sheets (Dental Prescale; GC) were used [[33], [34], [35], [36], [37], [38]]. These masticatory function values had already been measured in the previous conventional dentures and were recorded as baseline values.

**Fig. 5:**
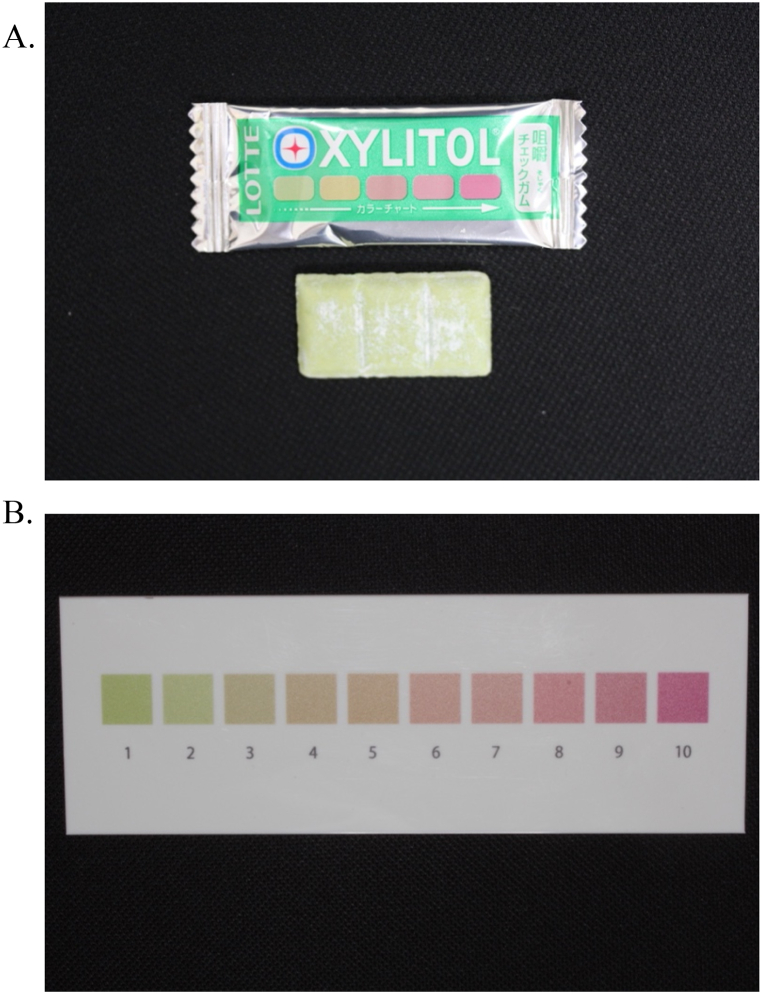
Color-changeable chewing gum. A, The gum is initially green and gradually becomes red on chewing. B, Color scale (Oral Care) from 1 (predominantly green) to 10 (predominantly red) points used for color-changeable chewing gum analysis. (For interpretation of the references to color in this figure legend, the reader is referred to the Web version of this article.)

The color-changeable chewing gum is initially green and gradually becomes red on chewing (Fig. 5A). It is used to evaluate the mixing ability [38,39]. The patients were allowed to freely chew the color-changeable chewing gum for 60 strokes before spitting it out. The color of the gum was visually evaluated using a color scale (Oral Care) ranging from 1 (predominantly green) to 10 (predominantly red) points (Fig. 5B). When the green and red colors were mixed, the average of the lowest and highest scores was considered the representative value.

**Fig. 6:**
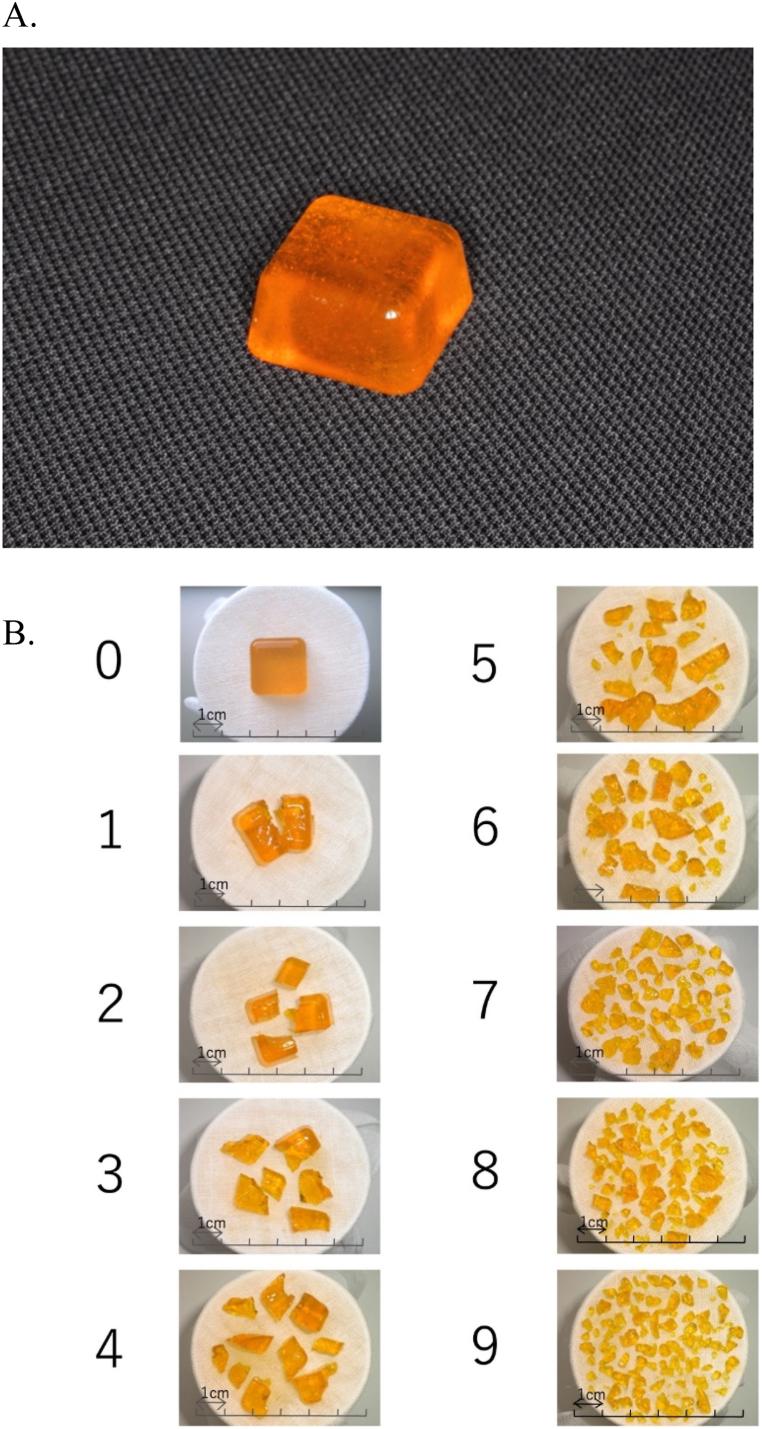
Gummy jelly (UHA Gustatory Sugar). A, It can measure masticatory function. B, Crushed state of gummy jelly pieces classified into ten levels (that is 0–9).

Gummy jellies that can measure masticatory function (UHA Gustatory Sugar) were used to evaluate biting ability (Fig. 6A) [33]. The masticatory function was measured by asking the participants to freely chew the gummy jelly 30 times and spit it onto a gauze stretched over a paper cup. A plastic spatula was used to unfold the crushed pieces of gummy jelly on the gauze placed over the paper cup. The crushed state of these gummy jelly pieces was classified into ten levels (that is 0–9) using a scoring method (Fig. 6B) [33].

**Fig. 7:**
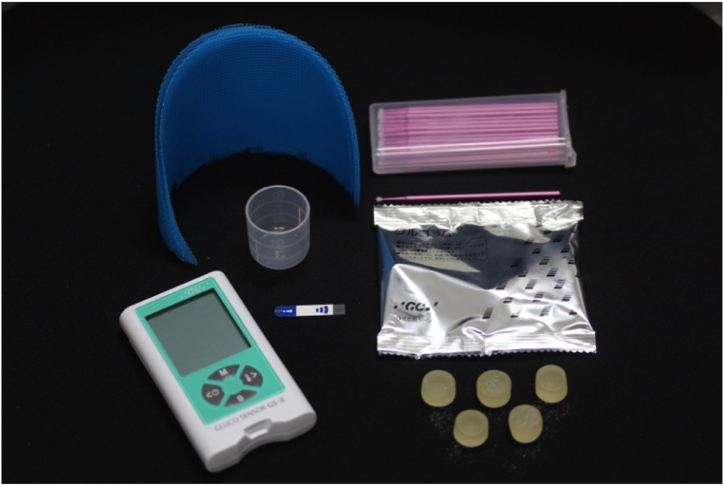
Gummy jelly (Glucolum, GC) and glucose-testing device (GS-II: GC).

Another gummy jelly (Glucolum; GC) was used to evaluate the masticatory function [39,40]. Patients were instructed to rinse their mouths with water before the measurement and chew the gummy jelly freely for 20 s. After chewing, the patients were instructed to hold 10 mL of water in their mouth and spit it out into a cup with a filter, and a solution containing glucose was collected. The solution was stirred, and a drop of this solution was used to measure the glucose concentration using a glucose-testing device (GS-II: GC) (Fig. 7).

**Fig. 8:**
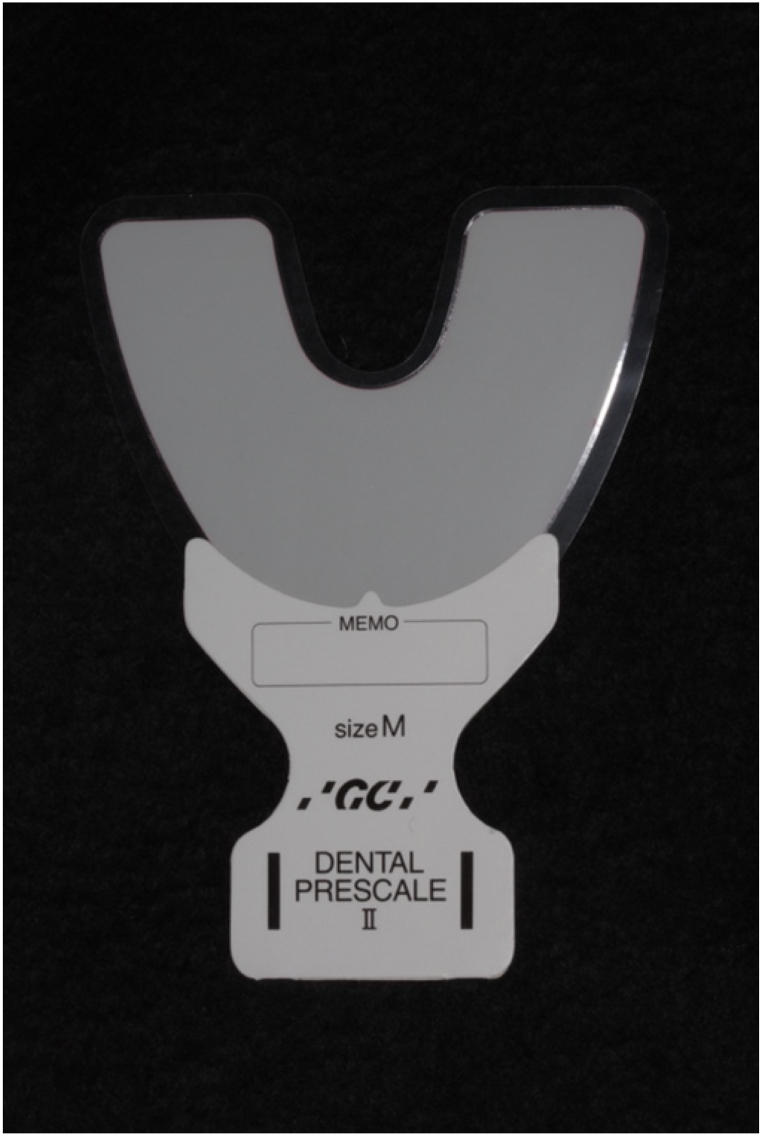
Pressure-sensitive sheets (Dental Prescale II; GC).

The maximum occlusal force and occlusal contact area were evaluated using pressure-sensitive sheets (Dental Prescale II; GC) (Fig. 8) [34]. An appropriately sized prescale was inserted into the participants’ mouth such that the entire dentition was placed on the film, and they were asked to clench their teeth for 3 s in centric occlusion. The occlusal force (N) and occlusal contact area (mm^2^) were then measured using the Occlusal Force Analysis System (GC).

### 3.1 Statistical analysis

The scores for masticatory function based on the color-changeable chewing gum and two kinds of gummy jellies, maximum occlusal force, and occlusal contact area of the patients with digital CDs fabricated using CDM were statistically analyzed using Steel test for multiple comparisons between baseline and 1 M, 6 M after the new dentures were delivered and adjusted. The significance level was set at 0.05. Statistical software JMP 17 (SAS Institute Inc.) was used.

## 4 Results

The baseline characteristics of the participants are shown in Table 1. The participants included 8 women and 12 men, with a mean age of 77.6 years. Table 2 presents the scores for masticatory function based on examinations with the color-changeable chewing gum and two kinds of gummy jellies, maximum bite force, and occlusal contact area at 1 M and 6 M.

**Table 1:** Baseline characteristics of the participants.

Age (years)	77.6 (63–87)
Sex (%)	
Female	8 (40 %)
Male	12 (60 %)

**Table 2:** Results of the color-changeable chewing gum, two kinds of gummy jellies, maximum bite force, and occlusal contact area analyses.

		Baseline (n = 20)	1 M (n = 20)	6 M (n = 18)
**Color-changeable chewing gum**	Mean	6.1	7.7	7.5
	SD	2.7	2.1	2.2
	Median	7.0	8.0	8
	IQR	[4.5, 8.0 ]	[7.0, 9.0]	[7.0, 9.0]
	P values		0.083	0.157
**Gummy jelly**	Mean	1.4	2.4	3.3
	SD	1.7	2.5	2.2
	Median	1.0	1.5	4.0
	IQR	[ 0.0, 2.0]	[0.0, 3.5]	[1.0, 5.0]
	P values		0.387	0.020*
**Glucolum (mg/dL)**	Mean	91.6	128.3	150.7
	SD	47.7	43.3	50.4
	Median	80.0	122.0	157.5
	IQR	[60.0, 106.8]	[99.3, 146.0 ]	[111.5, 185.8]
	P values		0.012*	0.003*
**Maximum bite force (N)**	Mean	290.6	304.2	263.0
	SD	134.6	155.0	177.7
	Median	310.5	255.6	220.0
	IQR	[190.6, 398.6 ]	[190.4, 427.0 ]	[ 120.0, 368.9]
	P values		0.989	0.616
**Occlusal contact area (mm2)**	Mean	12.0	10.9	11.5
	SD	6.3	5.7	6.2
	Median	11.5	8.7	11.7
	IQR	[6.8, 16.9]	[6.7, 15.6]	[8.1, 13.7 ]
	P values		0.820	0.966

Baseline is before the fabricating digital CD using CDM.

SD = standard deviation; IQR = interquatile range; M = month after delivered and the adjustment.

*Significant differences, P < .05 (Steel-test.).

The color-changeable chewing gum analysis indicated that there was no significant improvement of masticatory function from baseline to 1 M (P = .083) and 6 M (P = .157). The gummy jelly analysis indicated no significant difference between the scores at baseline and 1 M (P = .387); however, a significant improvement was observed from baseline to 6 M (P = .020). The test with Glucolum indicated a significant improvement in masticatory function from baseline to 1 M (P = .012). and 6 M (P = .003). Further, there was no significant improvement in the maximum bite force and occlusal contact area at 1 M or 6 M.

## 5 Discussion

The null hypothesis of this study was rejected for masticatory function assessed with the two kinds of gummy jellies but accepted for masticatory function measured with the other methods.

Many methods for assessing masticatory function exist, and each method reportedly has specific advantages, limitations, and relevance [35]. In the present study, color-changeable chewing gum and two types of gummy jellies were used to objectively evaluate different properties of masticatory function. Previous papers reported that different aspects of masticatory function respond differently to denture placement [36]. The color-changeable chewing gum method is simple, easily accessible, and effective. However, because it is soft and relatively easy to chew, it provides accurate results even in edentulous patients with reduced masticatory function [22]. In the current study, there was a trend of improvement in the masticatory function scores measured using the color-changeable chewing gum method at 1 month and 6 months after the delivery of the digital CDs; however, no statistically significant increase was observed. Furthermore, because the baseline values were high, no statistically significant increase was observed. Post-hoc analysis showed that the sample size of the current study was appropriate as the statistical power for detecting differences in color-changeable chewing gum at 1 month after the delivery was 0.79. Previous studies revealed that the mixing ability evaluated using color-changeable chewing gum correlates with the area of occlusal contact [37]. Therefore, as observed in the present study, since the existing CDs have more occlusal wear on the artificial teeth and more occlusal contact area, the baseline scores tend to be higher, and ceiling effects are likely to be observed on using color-changeable chewing gum for evaluation. This finding is consistent with the results of a previous study [36] and may explain the lack of a significant improvement at a relatively early stage after denture fabrication in the present study.

On the other hand, gummy jelly is more elastic than gum and is a harder chewing substance. It may be too hard or bulky for edentulous patients with reduced masticatory function [34]. Furthermore, patients with removable dentures, such as the participants in the current study, may find it more difficult to chew gummy jelly than gum because the artificial teeth have compromised anatomical morphology and the patient has reduced muscle strength. However, a significant increase in the gummy jelly score was observed at 6 months after placing the new dentures in the present study. In this study, the mean masticatory function score at 6 months using gummy jelly was 3.3 and that with Glucolum was 150.7 (mg/dL), which were similar to the scores of 2.0–3.0 reported previously [39] and 180.3 (mg/dL) [40] with conventional CDs. The gummy jelly test is reportedly significantly correlated with the occlusal force and occlusal contact area [38], and community ability is likely to be influenced by teeth and masticatory muscles. The increase in the gummy jelly score in the present study suggests that digital CDs fabricated using CDM contribute to good recovery of the masticatory function in elderly patients with edentulism.

The maximum bite force of 310.5 [190.6, 398.6] N at baseline in the current study was considerably higher than the average maximum bite force of 220 N among completely edentulous patients reported previously [34]. However, the values of 255.6 [190.4, 427.0] N at 1 month and 220.0 [120.0, 368.9] N at 6 months after the delivery of the new dentures were similar to those reported previously. The slightly lower values than those with the old dentures could be attributed to an "adaptation" issue immediately following denture delivery. In general, approximately 3 months are needed to adapt to a denture [41]. The participants in the current study were elderly, with an average age of 78 years; therefore, it may have taken them some time to adapt to the new dentures. The CDM used in this study is characterized by good adhesion between the denture base and artificial teeth and the possibility of using ready-made hard resin teeth with excellent wear resistance [18].Therefore, CDs fabricated with CDM are less resistant to occlusal wear and can maintain an appropriate occlusal height for a prolonged duration. On the other hand, the increase in the occlusal contact area and subsequent recovery of occlusal force are affected by the increase in the occlusal contact area of the molars [28]. Therefore, as with conventional full dentures, recovery of occlusal forces may not be observed immediately after the delivering the digital denture and may occur later as the artificial teeth are worn by abrasion.

Furthermore, color-changeable chewing gum and gummy jelly were used to evaluate the masticatory function in the present study. However, the results may differ on using other test foods or masticatory materials. Thus, a subsequent subjective evaluation of digital CD made using CDM is planned.

## 6 Conclusion

Based on the findings of this prospective clinical study, the following conclusions were drawn.

1. The digital denture fabricated using CDM results in good recovery of the masticatory function in elderly patients with edentulism.
2. Furthermore, the present findings combined with the cost-effectiveness and patient satisfaction associated with CDM indicate that it is a suitable denture fabrication method in clinical settings.

## Patient consent

Written informed consent was obtained prior to this clinical study from all participants.

## Data availability statement

The data presented in this study are available on request from the corresponding author.

## Additional information

No additional information is available for this paper.

## CRediT authorship contribution statement

**Maiko Iwaki:** Writing - original draft, Visualization, Project administration, Methodology, Investigation, Funding acquisition, Data curation, Conceptualization. **Manabu Kanazawa:** Supervision, Project administration, Investigation, Formal analysis, Conceptualization. **Yumika Soeda:** Validation, Software, Investigation. **Tamaki Hada:** Investigation. **Yuriko Komagamine:** Validation, Investigation. **Shunsuke Minakuchi:** Validation, Supervision.

## Declaration of competing interest

The authors declare the following financial interests/personal relationships which may be considered as potential competing interests:

Maiko Iwaki reports financial support was provided by JSPS, Japan; 10.13039/501100001691Japan Society for the Promotion of Science. If there are other authors, they declare that they have no known competing financial interests or personal relationships that could have appeared to influence the work reported in this paper.
